# Investigations on Cavitation Erosion and Wear Resistance of High-Alloy WC Coatings Manufactured by Electric Arc Spraying

**DOI:** 10.3390/ma18102259

**Published:** 2025-05-13

**Authors:** Edmund Levărdă, Dumitru-Codrin Cîrlan, Daniela Lucia Chicet, Marius Petcu, Stefan Lucian Toma

**Affiliations:** 1 Department of Materials Engineering and Industrial Safety, Faculty of Materials Science and Engineering, Gheorghe Asachi Technical University of Iasi, 700050 Iasi, Romania; levarda.edmund@gmail.com (E.L.); dumitru-codrin.cirlan@student.tuiasi.ro (D.-C.C.); stefan-lucian.toma@academic.tuiasi.ro (S.L.T.); 2 Materials Science Department, Materials Science and Engineering Faculty, Gheorghe Asachi Technical University of Iasi, 700050 Iasi, Romania

**Keywords:** arc spraying process, coating, erosion by cavitation, dry friction wear

## Abstract

Due to the low hardness of carbon steels, their low resistance to wear, and erosion by cavitation and corrosion, it is necessary to protect the surfaces of parts with layers capable of ensuring the properties listed above. In this paper, we started from the premise that adding tungsten carbide (WC) powders during the electric arc spraying process of stainless steel would lead to obtaining a composite material coating resistant to wear and erosion at high temperatures, with relatively lower manufacturing costs. Thus, our research compared the following two types of coatings: a highly alloyed layer with WC, Cr, and TiC (obtained from 97MXC core wires) and a 60T/WC coating (obtained from a 60T solid-section wire to which WC was added), in terms of microstructure, mechanical properties, dry friction wear, and behaviour at erosion by cavitation (EC). The results of our research demonstrated that although the 60T/WC coating had lower erosion by cavitation behaviour than the 97MXC one, it can still be considered as a relatively good and inexpensive solution for protecting C15 steel parts.

## 1. Introduction

Recognised as a very important problem due to the damage caused, the phenomenon of erosion by cavitation (EC) of carbon steels requires increased research efforts by numerous research groups to extend their field of application to turbulent liquid environments [1,2,3]. It is known that with modification of the hydrostatic pressure at the metal–liquid interface, vapour cavities appear. The increase and subsequent sudden decrease in the volume of these bubbles produce shock waves within the contact surface, which determine local increases in the temperature and pressure on small areas (10^−10^ m^2^) in a relatively short period (<1 μs) [4,5,6]. On stainless steel, brass, and aluminium surfaces subjected to cavitation phenomena, needle-shaped erosion pits appear, which grow in size up to several square millimetres [7,8,9]. In the case of carbon steels, the adjacent surfaces of the EC pits are circular, in the form of a ring [10]. The low hardness of carbon steels [11,12] and the presence of various reactive ion species in solution determine the increases in the ring’s dimensions and the appearance of irregular oxide structures on its surface [13,14], which irreparably damage the surfaces of parts. Due to its low price, carbon steel is widely used in many fields, but its resistance to EC and unsatisfactory corrosion limits its applicability [15,16]. Stainless steels, which exhibit a high hardness and corrosion resistance due to the presence of chromium, have attracted attention for use in environments exposed to EC and corrosion [17,18,19]. However, the high prices of stainless steels and their relatively acceptable hardness mean that they are not used in highly aggressive hydraulic environments [20]. By comparing the characteristics of carbon steels, including their advantages and disadvantages, different research groups have proposed reproducible, durable, and economical solutions, expanding, in this way, the area of applicability of carbon steels [3,21]. It is known that coatings obtained by thermal spraying are widely used to improve the anti-wear properties of components subjected to hostile operating conditions. However, coatings with a high tungsten carbide and chromium content obtained by thermal spraying techniques may constitute promising alternatives to various technological solutions for obtaining wear-, EC-, and corrosion-resistant deposits, as the environmentally unfriendly EHC (electrolytic chromium plating). Deposits of highly alloyed metallic composite materials with Cr and WC, called cermets [22,23,24], obtained by thermal spraying in an electric arc, constitute relatively cheap and viable alternatives for obtaining parts resistant to wear and erosion by cavitation (pump components, valves, propeller blades, etc.). The properties of coatings incorporating cermet, including dry wear resistance, in different technological conditions, may vary depending on the properties of the raw material, the method of embedding the hard particles in the metal matrix [25], the chosen spraying process [26], and the technological parameters used in the deposition process [27,28].

It is known that carbon steels are characterised by a low resistance to wear and erosion by cavitation and corrosion, which is why it is necessary to improve the properties of the superficial layer by different technological means, such as thermal treatment, chemical conversion, physical vapour deposition, and thermal spraying. In this paper, high-alloy steel coatings with Cr and WC were deposited by thermal spraying in an electric arc on low-alloy steel substrates (C15) in order to obtain a layer resistant to dry friction, wear, and cavitation erosion. Thus, our research compared the physical, chemical, and mechanical properties of the following two coatings: a highly alloyed coating with WC, Cr, and TiC obtained from 97MXC core wires and a classic Cr-alloyed coating obtained from 60T solid-section wire to which WC was added—symbolically called 60T/WC. Thus, we started from the premise that the addition of tungsten carbide to a material containing a significant amount of chromium would lead to an increase in resistance to dry friction wear and an increase in resistance to EC. By using thermal spraying in an electric arc, we wanted to improve both the wear and EC resistance of the low-alloy steel substrate (C15), knowing that these two factors are responsible for the efficiency and reliability of some parts [29,30].

## 2. Materials and Methods

**Figure 1:**
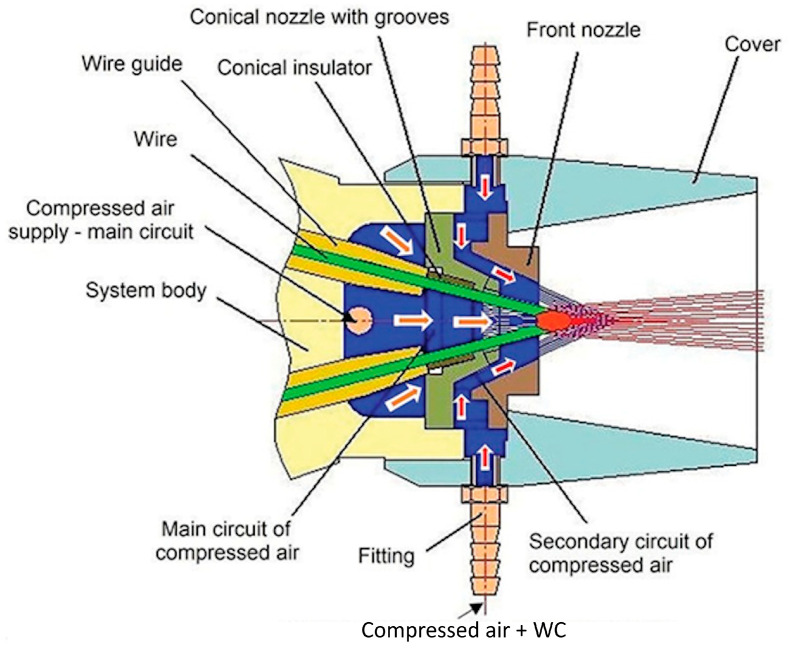
Nozzle system used to obtain 60T/WC coating [31].

**Figure 2:**
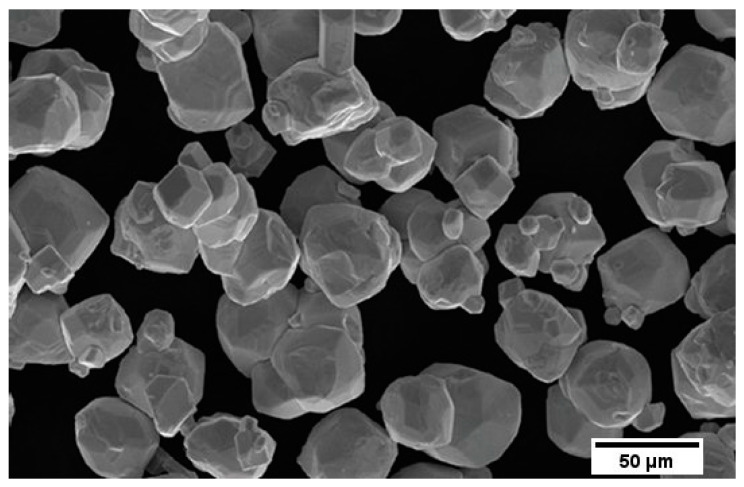
Tungsten carbide powder micrograph.

In the framework of our research, we used C15–EN10083 steel samples (with dimensions of 60 × 60 × 15 mm) coated with layers of high-alloy 97MXC steel and layers of 60T/WC (60T mixed with tungsten carbide). The 97MXC and 60T/WC layers were deposited using Tafa CoArc System thermal arc spraying apparatus, equipped with a model 9930 spray gun—both manufactured by Tafa Praxair, Road, Concord, NH, USA. For the deposition of the 60T/WC layers, the 9930 spray gun was equipped with a spraying nozzle system—an original design that allowed for the introduction of tungsten carbide powder into the electric arc. The WC powder was introduced at a controlled rate of approximately 20 g/min, ensuring reproducibility and a consistent particle flow during spraying. Figure 1 presents the spray nozzle system used for obtaining the 60T/WC layer. It is formed by an assembly of convergent conical nozzles that ensure a convergent–divergent geometry of the compressed air jet. The following two compressed air circuits are formed in the space between the concentric nozzles and the body of the atomising system: (a) a primary compressed air circuit (orange arrow), which has the role of breaking the liquid droplets from the surface of the contacting wires and dispersing the droplets into fine particles [31] and (b) a secondary compressed air circuit, which has both the role of constricting the arc (due to the inclination angle of the inner surface of the front nozzle) and the role of transporting fine WC particles into the arc zone (red zone). The two compressed air circuits are fed from two different sources with different pressures [31]. The spraying process was conducted under controlled environmental conditions, at a temperature of 22 ± 2 °C and relative humidity of 40 ± 5%, to minimise variability in coating quality. The materials used were tubular wire (97MXC—diameter 1.6 mm) or solid wire (60 T—diameter 1.6 mm) manufactured by Tafa Praxair, USA, whose commercial chemical compositions are presented in Table 1. For the addition, a tungsten carbide (WC) powder was used, with a grain size between 15 and 120 µm, produced by Titan International Inc. (Pottstown, PA, USA), as presented in Figure 2. The choice of the spray parameters (current, voltage, pressure air compressed, and SOD) was based on performing some preliminary optimisation tests that followed the stability of the electric arc and the atomisation and acceleration of the particles, so as to obtain the best adhesion and minimum porosity of the coating [32,33].

The C15 steel samples, after they were cut, were mechanically cleaned by corundum blasting, with a grain size of EKF 14, at a pressure of p = 4 bar. Then, they were chemically cleaned by washing with ethanol in an ultrasonic bath to eliminate traces of grease and any corundum particles fixed on the steel surface. Immediately after they were chemically cleaned, the samples were coated with a layer of 97MXC or 60T/WC by electric arc thermal spraying. Table 2 presents the parameters of the thermal spraying process in the electric arc. All tests were performed with at least three replicates per experimental condition to ensure the reproducibility and reliability of the results. The data obtained were statistically analysed, reporting the standard deviation for critical measurements.

The selected method (electric arc spraying) was compared to alternative methods (HVOF and plasma spraying), demonstrating advantages such as lower operational costs and efficient deposition rates for large-area coatings [32]. Safety protocols for operator protection (personal protective equipment and fume extraction systems) and environmental considerations (waste management and disposal) were rigorously applied during the experiments.

In order to characterise the microstructural and morphological characteristics of the coatings obtained by thermal spraying, the samples were cut in the transverse plane, mounted in conductive resin, ground, and then polished in a cross-section, according to standard metallographic procedures. The microstructure of the sprayed layers was examined on the cross-sections of the polished samples, using the Secondary Electrons mode (ETD—Everhart–Thornley Detector) of a scanning electron microscope (Vega II LSH, Tescan—Brno, Czech Republic), while the chemical composition was analysed by EDS (energy-dispersive X-ray spectrometer). The EDS measurements were performed in randomly distributed areas of the coating. The thickness of the coatings was also investigated using SEM. The roughness of the deposits was determined using an SJ301 roughness meter (made by Mitutoyo, Kawasaki, Japan) according to the ISO 21920-3:2021 standard [34]. The porosity of the coatings was estimated according to the ASTM E 2109-01/2021 standard [35], and was calculated using the IQ Materials 2.1 image analysis software (manufactured by Media Cybernetics, Rockville, MY, USA) [32] and SEM micrographs. The phases and constituents within the deposits were determined by X-ray diffraction using the X’PERT PRO MRD diffractometer (Panalytical, Almelo, The Netherlands), with the following working configuration: Cu anode with λ = 1.54 Å, open Eulerian cradle sample support, and 2θ = 20–90° [36]. Calibrations of the analytical instruments (SEM, EDS, microhardness tester, analytical balance, and profilometer) were performed according to the manufacturers’ recommendations before experimental use.

**Figure 3:**
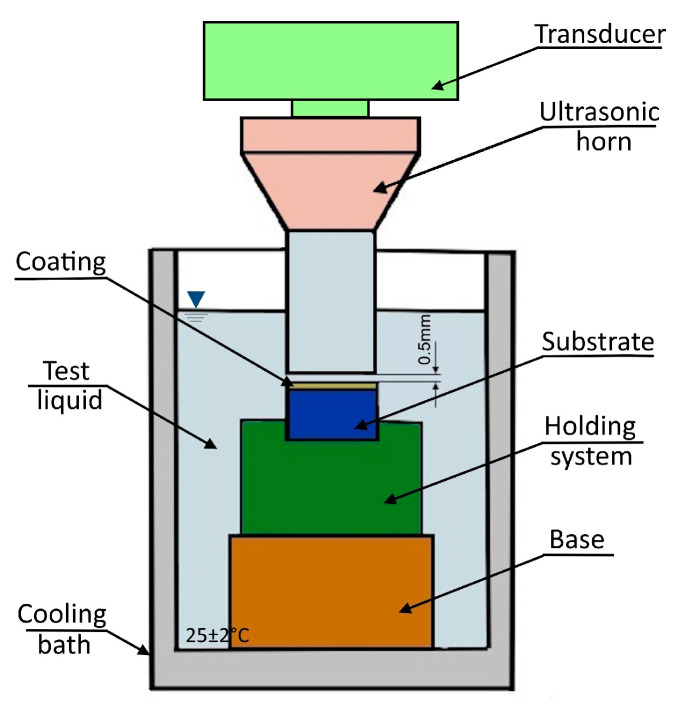
The schematic of the ultrasonic vibration system used for cavitation study.

To evaluate and compare the erosion resistance observed through the cavitation of the coatings obtained by thermal arc spraying and that of the substrate, we used an ultrasonic vibration system and test conditions described in previous works [37,38]. In this case, the cavitation phenomenon was generated by a magnetostrictive ultrasound device, which worked at a resonant frequency of 20 kHz and developed the resonant amplitude from a peak-to-peak of 50 μm, in accordance with the ASTM G32-16(2021)e1 standard [39,40]. Before testing, the square surface of the samples (25 mm × 25 mm) was ground and polished to a roughness level of Ra < 0.6 μm, and the distance between the ultrasonic probe and the sample surface was 0.5 ± 0.05 mm. The measurements were performed using the method of stationary sample, the total duration of the test was 6 h, performed at 1/2 h intervals, and the samples were weighed with an analytical balance with a precision of 0.01 mg. To calculate the volume losses, we considered the following experimentally determined densities, according to the ASTM B962-17 standard [41]: $\rho_{97MXC}=8.32\;g/{cm}^{3}$, $\rho_{60T/WC}=7.22\;g/{cm}^{3}$, and $\rho_{C15}=7.85\;g/{cm}^{3}$. Thus, the average erosion depth, the variation in the erosion rate over time, the maximum erosion rate, and the incubation period could be determined. The investigation’s cavitation erosion results for the 97MXC and 60T/WC coatings were compared with the C15 substrate material, which was used as the reference sample. Surfaces worn by erosion through cavitation were examined with SEM-EDS. A schematic of the ultrasonic vibration system used for the erosion by cavitation study is presented in Figure 3.

The microhardness of the deposits was determined by Vickers indentation, using the CV-400 microhardness tester (produced by CV—Instruments, Tokyo, Japan) equipped with a Vickers pyramidal indenter and a 300 g load, which acted on the transverse surface of the deposits for 10 s, according to the ISO 6507-1 standard [42,43,44]. Due to the large variations between the recorded microhardness values, 20 measurements were performed on each coating, and the average values are reported.

The coating’s adherence to the substrate was determined by tensile testing, in accordance with the ASTM C633-13 (2021) [45]. Using an Amsler-type tribometer, the wear behaviour of 97MXC and 60T/WC coatings was evaluated according to the ASTM G32 standard. Before wear resistance was evaluated, the 97MXC and 60T/WC coatings were sanded with abrasive paper with different granulations (P100, P500, P800, and P2000) and polished with a diamond suspension that had a granule size of 3 μm. The wear resistance tests were performed on steel samples (C15) covered with 97MXC or 60T/WC, respectively, with dimensions of 20 × 40 × 15 mm, in a dry friction regime for 1 h, using a rotation speed of 100 rot/min and a force of 20N and 40N. In order to evaluate the sliding system formed, we analysed the friction moment developed during the tests, in the coating—counterproof contact area, as Dorner-Raser et al. proceeded [46]. In wear tests typical for adhesives, both the coefficient of friction and the frictional time have been related using the following relationship [24]:

γ = M/(F∙R) (1)

where γ is the coefficient of friction, F is the applied force, R is the radius of the approved disc, and M is the friction moment.

Images of the wear traces in both the transverse section and along the wear marks were investigated with the help of a Taylor–Hobson profilometer and by scanning electron microscopy (SEM) coupled with an EDS (energy-dispersive X-ray spectrometer) device.

## 3. Results

**Figure 4:**
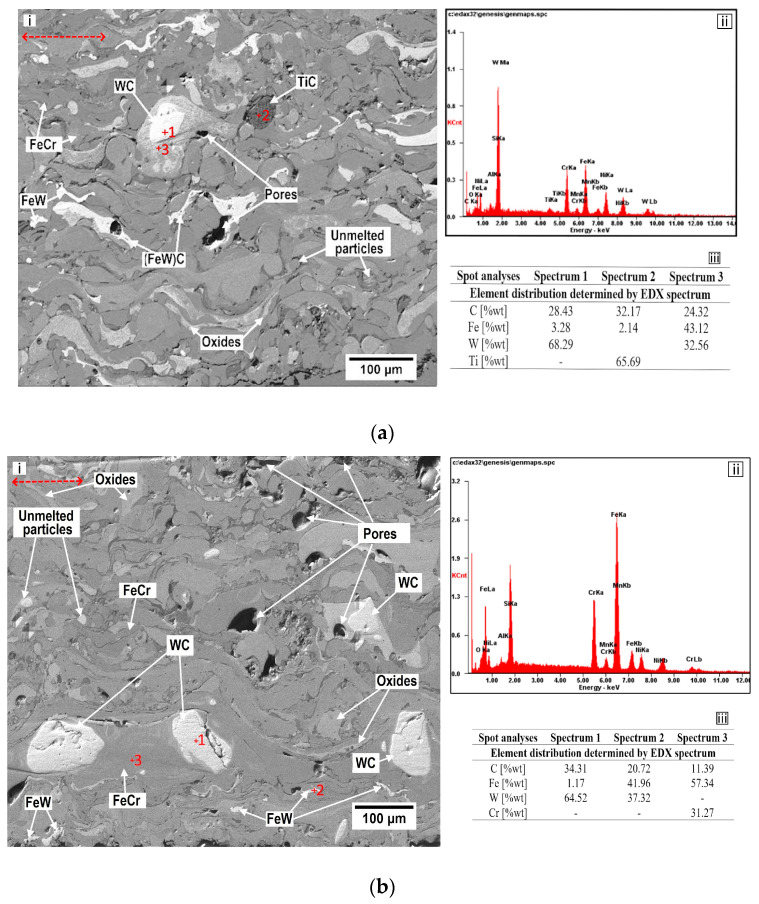
SE (Secondary Electron) images (i), EDX spectra (ii) and chemical elements values (iii) on the transverse section of (**a**) 97MXC and (**b**) 60T/WC coatings.

Figure 4 presents SEM micrographs of the transverse sections of the 97MXC and 60T/WC coatings obtained under conditions using the process parameters presented in Table 2. The images in Figure 4 were processed at maximum contrast (black = 0 and white = 255), and the dominant metal phases correspond to grey level 150, which allows for the identification of pores, oxide phases, and metal carbides. It can be observed that the coatings present a heterogeneous microstructure, formed of lamellas oriented parallel to the substrate surface, partially melted or unmelted spherical particles, polygonal formations, and pores. It can be affirmed that the microstructures presented in Figure 4a,b are specific to the layers obtained by the electric arc thermal spraying method. The appearance of partially melted or unmelted particles in the coating can be caused by both the long residence time of the jet by the sprayed particles and by the rapid cooling of some chemical compounds with high melting temperatures. Both causes lead to the formation of pores and cracks inside the layer [47], which reduces durability by propagating such cracks or due to the appearance of the delamination phenomenon—aspects also reported by Hong S. et al. [47]. The average thicknesses of the deposits obtained by thermal spraying in the electric arc were located in the range of 956 ± 26 μm for 97MXC and 884 ± 45 μm for 60T/WC. In Figure 4(a-ii,a-iii,b-ii,b-iii), the energy spectra and chemical compositions at different points on the cross-sections of the coatings are presented. The data in Figure 4(a-ii) confirm the presence of the elements W, Ti, B, and Cr in the deposit of 97MXC and the presence of the elements W, C, Cr, and Ni in the 60T/WC coating, respectively—see Figure 4(b-ii). In Figure 4(a-i), it can be seen that WC and TiC particles, presented inside the wires, were completely melted or partially melted in the electric arc. Comparatively, in the 60T/WC coating—see Figure 4(b-i)—it can be observed that the WC particles inserted in the arc were found unmelted in the layer, preponderant in the polygonal form. In both coatings, the metal binder (Fe) was completely melted and was distributed around the unmelted WC or TiC particles, similar to the results reported by Tillmann et al. [25]. The red arrow dashed line in Figure 4(a-i,b-i) represents the spray direction.

**Figure 5:**
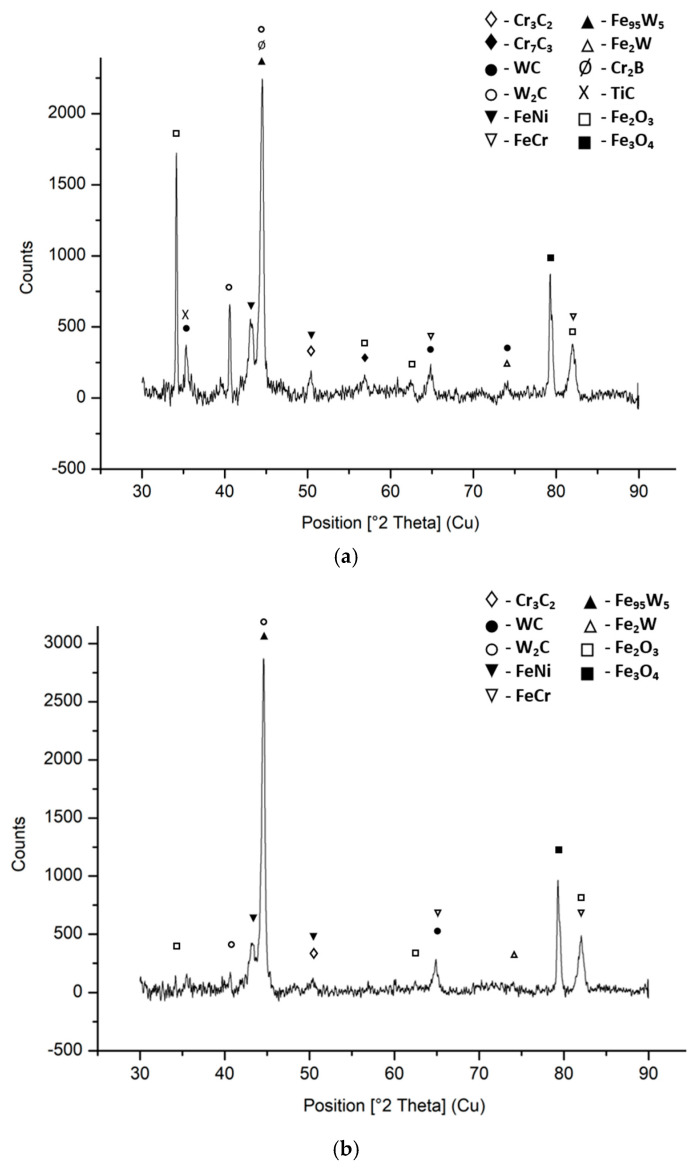
XRD pattern as sprayed: (**a**) 97MXC coating and (**b**) 60T/WC coating.

In Figure 5, the XRD diffraction patterns of 97MXC and 60T/WC are presented, obtained on polished surfaces using Cu-Kα radiation. It can be observed that both coatings contain Fe-Cr alloy as iron oxides of the Fe_2_O_3_ and Fe_3_O_4_ type, as well as WC and W_2_C peaks resulting from the decomposition, during the thermal spraying process, of WC in unique elements, C and W_2_C, similar to the reported results and other research groups with similar concerns [48]. Also, it can be seen that the magnitude of the W_2_C peaks is higher in the diffraction model of 97MXC compared to the model of 60T/WC—see Figure 5a vs. Figure 5b. This aspect suggests that during spraying in the electric arc of the 97MXC layer, the phenomenon of WC decomposition is more intense. The diffraction model of the 97MXC coating contains additional titanium carbide, solid alloy solutions of γ(Fe, Ni) and γ(Ni, Cr), complex carbides of the FeW_3_C, Fe_3_W_3_C, and Fe_6_W_6_C type, and FeB and Cr_2_B fractions.

The SEM micrographs, EDX analyses—Figure 4(a-ii,b-ii)—and XRD patterns—Figure 5—allow for the identification of different phases of the coatings. Thus, it is observed that the matrices are composed of light grey metal splats associated with the FeCr and FeW phases. At the edge of the flattened surfaces, certain interstitial oxides are formed, and the areas with closed contrast indicate the presence of pores.

The porosity of the 97MXC and 60t/WC coatings is typical of steel deposits obtained by thermal spraying in an electric arc. Thus, in Table 3, the average values of porosity calculated for the two deposits are presented. It is found that the 97MXC layers, despite containing a larger amount of high melting points, have a lower porosity than the layers of 60T/WC. This aspect can be explained by the fact that, in the process of obtaining the deposits of 97MXC, the hard fuse chemical compounds (WC, TiC, borides, and chrome carbs) melted in the electric arc with the wire, while in the process of obtaining 60T/WC, the Wolfram carbides were introduced in the zone of the electric arc.

The two deposits presented values of close average adhesion, a fact which suggests that the value of the adhesion of the two composite materials is given by its matrix of iron. The higher average adhesion values of the deposits of 97MXC vs. deposits of 60T/WC are due to the difference between the average porosity of the two coatings, similar to the results reported by Haraga R.A. et al. [33]. The presence of hard chemical compounds in the 97MXC deposits explains the significant differences in microhardness between the two coatings—see Table 3, similar to the results reported by He D.G. et al. [48].

In this study, samples coated with 97MXC or 60T/WC were subjected to erosion by cavitation, by applying relatively high cavity loads, which represent the amplitude of the vibration, and the distances were 50 μm and 0.5 mm, similar to the reports of Jonda E. et al. [50]. In this study, the resistance to erosion by cavitation of the substrate was considered, which could influence the erosion resistance of coatings. The highest erosion resistance by cavitation is given by the layer that presents the smallest volume loss. In Table 4, the results obtained after the samples were subjected to erosion by cavitation are presented.

**Figure 6:**
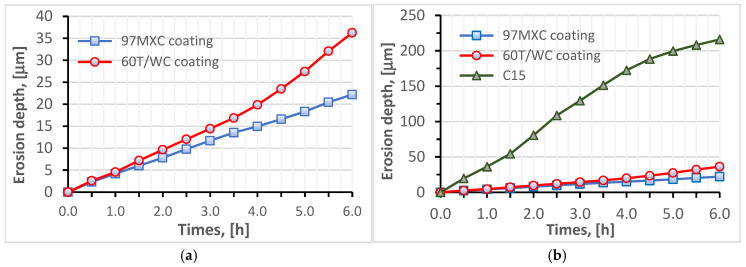
The average values of the depth of erosion (MDE) over time, estimated for (**a**) 97MXC and 60T/WC deposits and (**b**) C15 steel substrate vs. 97MXC and 60T/WC deposits.

**Figure 7:**
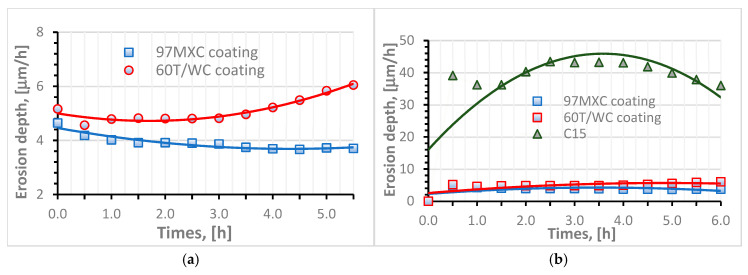
Erosion rate–time curves estimated for (**a**) 97MXC and 60T/WC deposits and (**b**) C15 steel substrate vs. 97MXC and 60T/WC deposits.

As it can be seen from Figure 6, the 97MXC coating had MDE = 22.2 µm after 6 h of testing, while the 60T/WC layer presented comparative values after 4.5 test hours (see Figure 7a) and the steel substrate C15 immediately after 30 min of cavitation exposure (see Figure 7b). Therefore, we can say preliminarily that the 60T coating can be a successful, economic alternative to improve the erosion resistance to cavitation of C15 carbon steel.

Figure 7 presents the erosion by cavitation rates of the 97MXC and 60T/WC coatings, as well as that of the substrate material. It can be seen from the graph in Figure 7a that both coatings present a negligible erosion incubation period and a “cleaning effect” that manifests itself in the first hour of testing. This phenomenon refers to the effect of removing particles from the surfaces of coatings obtained by thermal spraying—an aspect also observable in the work of other researchers [30,51]. In addition, the two coatings behaved differently in the erosion by cavitation testing. The 97MXC layers showed a variable erosion rate up to 2 h of testing, after which it stabilised around an average value of 3.9 µm/h. The erosion rate of 60T/WC deposits was almost constant at 4.8 µm/h until the third hour of testing, after which it increased to 6.04 µm/h—recorded in the sixth hour of testing. Compared to the erosion rates of the 97MXC and 60T/WC coatings, the erosion rate of the steel samples was higher—see Figure 7b. It increased to a value of 43.46 µm/h in the first 2.5 h of testing, remained constant around a value of 43.10 µm/h, then decreased to a value of 36 µm/h after 6 h of testing.

Our main findings were as follows:

- The 60T/WC coating presented a resistance to EC less than 1.67 times that of the resistance to EC of the 97MXC layer (see Table 4);
- After 1.5 h of testing, the 60T/WC coating had a stable erosion rate (approximately 4.8 µm/h);
- After 6 h of testing, the 60T/WC layer had an MDE of 1.6 times higher than that of 97MXC;
- Both coatings presented an average erosion depth at least six times lower than of the C15 steel sample.

From Figure 7b, it can be seen that the erosion rate of the C15 steel varies according to a curve describing an erosion pattern consisting of the following two phases: one of acceleration and the second of stabilisation. Based on the reports of Brijesh, V. et al. [11], it can be stated that, initially, the steel surface undergoes severe degradation due to the cavitation phenomenon, which causes microcrack nucleation in the area of crystalline defects, grain boundaries, or inclusions. As the exposure of the steel to cavitations continues, microcracks coalesce and detached steel fragments (known as micro-pitting) appear, the material loss becomes rapid, and the maximum erosion rate is reached at this stage. After the removal of the superficial layer, a strain-hardened substrate remains, which exhibits impact resistance, reducing the efficiency of cavitation. On the other hand, the pits and craters created by surface erosion disperse the energy produced by the collapsing air bubbles, directly reducing the impact. This phenomenon leads to the occurrence of the “stabilisation phase”—characterised by a low erosion rate—in agreement with the information available in the specialised literature.

As can be observed, the erosion rate of the 97MXC coating starts to increase after 2 h of testing and reaches a maximum of 3.32 μm/h after 5 h of exposure. It is further observed that in the sixth hour of the test, it has a decreasing trend, which shows that the coating maintains its integrity, exhibiting only a late minor degradation. Since the erosion rate for the 97MXC coating is the lowest, we can state that the layer has a negligible incubation period or even shows the longest incubation period (delay of erosion onset). It can be suggested that the matrix of the 97MXC composite layer, consisting of nickel-alloyed ferrite and hard particle content, shows a high toughness capable of allowing the absorption of micro implosion shocks without decohesion, i.e., without material loss. It is only after longer exposure that any fine cracks appearing at the carbide–matrix interface or within the matrix cause the gradual detachment of fragments and a slight increase in the erosion rate.

Compared to C15 steel, the 60T/WC coating exhibits a much lower erosion rate throughout the test, with a relatively small variation ranging from 4.8 to 6.04 μm/h for 6 h. This consistency suggests a very good stability of the coating over time, which means that the material resists cavitation without a progressive increase in deterioration. Practically, after a slight initial wear, the 60T/WC layer reaches an almost constant and low erosion regime. It can be suggested that the 60T/WC coating contains a hard stainless-steel matrix reinforced with tungsten carbides, which, although less ductile than a nickel one, still offers a high stiffness. In the first moments of exposure, the steel matrix yields those soft or slightly adherent fragments (hence, the low erosion rate observed immediately), but due to the high carbide content, wear is limited. The behaviour difference between the 97MXC and 60T/WC coatings, graphically highlighted (see Figure 6a and Figure 7a), has the following significance: the 97MXC coating prioritises the toughness of the matrix, i.e., it has almost a zero erosion rate at the beginning, after which it increases slightly (probably due to the appearance of microcracks), while the 60T/WC coating relies on the high intrinsic hardness of the matrix, i.e., it has a very low erosion rate at the beginning, after which it remains almost constant.

Even at the maximum reached by the erosion rates of the two coatings, the values recorded are very low compared to those of the C15 steel. Thus, it can be stated that a metal layer hardened with WC particles, well-embedded in the metal matrix, is characterised by a low erosion rate when exposed to EC, i.e., erosion progresses very slowly. These observations correspond with reports in the literature, which show that the addition of hard carbides to a protective coating enhances resistance against cavitation erosion both by increasing hardness and by improving surface toughness [52].

It may be suggested that although the 60T/WC layer exhibits behaviour to erosion by cavitation inferior to that of 97MXC deposition, it can be considered as a relatively good and cheap solution to protect the C15 steel parts.

**Figure 8:**
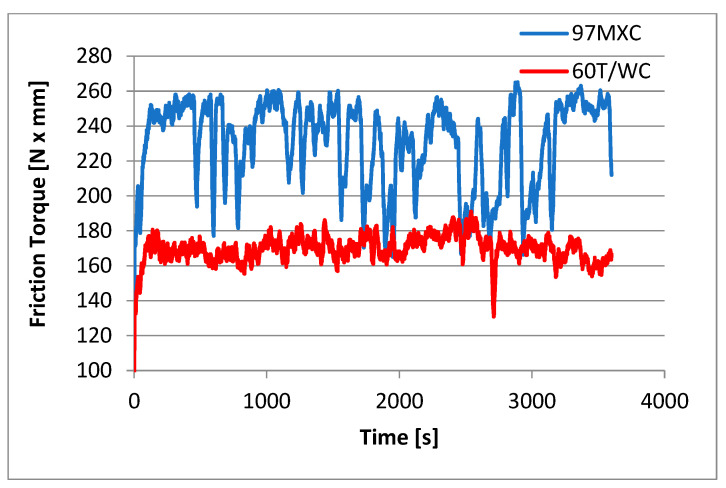
Variation in the frictional moment in time for a load of 40 N.

Although both coatings contained significant amounts of WC, they still behaved differently at slip. Thus, although the average friction coefficient value of the 97MXC deposits was higher than the average friction coefficient value of 60T/WC, the wear rate of the first deposit was lower than the wear rate of the second layer. It can be suggested that the higher friction coefficient value of coatings of 97MXC and their lower wear rate were due to the presence of the complex carbide and borides inside the TiC layer. Figure 8 presents the variation in time of the friction moment for a load of 40 N in dry friction conditions.

It can be seen that the frictional moment of the couple containing the 60T/WC layer is lower than the frictional moment of the couple containing the 97MXC coating (Table 5). It is noted that the friction moment of the couple containing the 60T/WC deposit presents relatively small variations in time, compared to the friction moment of the couple containing the 97MXC deposit. This aspect can be explained based on the presence of hard chemical compounds in the basic matrix of the 97MXC deposits—an aspect that provides a superior wear resistance compared to layers in which the binder is formed from an iron matrix alloyed with chromium. Similar results were reported by Ozkavak H and al. [53], where they compared the wear resistance of WC-CoCr and WC-Co coatings obtained by high-velocity thermal spraying (HVOF) on steel substrates.

**Figure 9:**
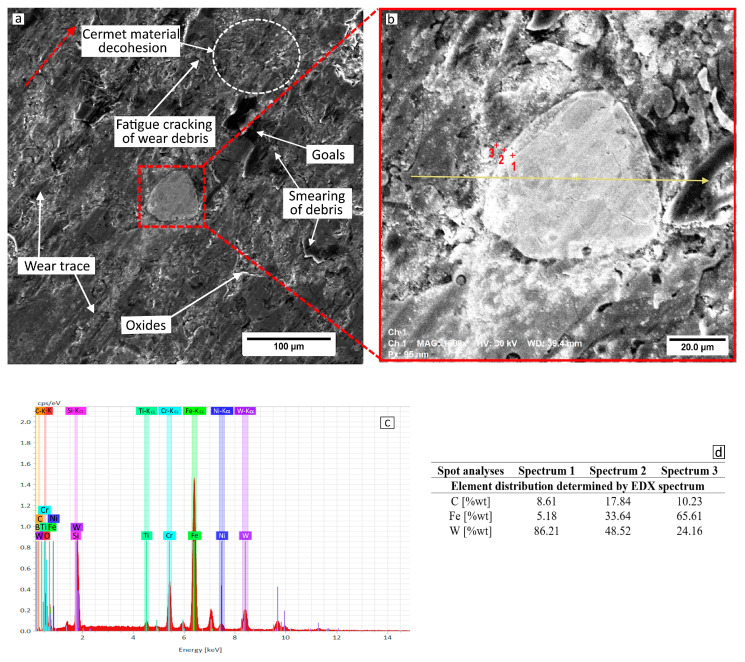
Surface damage of 97MXC: (**a**) SE image of the wear trace; (**b**) WC—detail (MAG: 1008x, HV: 30 kV, WD 39.4 mm); (**c**) spectrum of chemical elements in the median zone of tungsten carbide; and (**d**) EDX spot analysis.

An examination of Figure 9 shows that both coatings exhibit variations in frictional moment. This behaviour can be explained by the specific nature of dry friction, in which the absence of lubricant forces the surfaces in contact to interact directly through their roughness. This direct interaction favours the occurrence of characteristic phenomena such as local adhesion, the formation of hot micro-welds, material transfer between surfaces, and the formation of tribochemical oxide films. In addition, some types of abrasive or oxidative wear may also occur. According to studies conducted by Myalska H. et al. [54], two essential factors influencing the behaviour under these conditions are the hardness of the coating and the stability of the oxide film formed during friction.

The 97MXC coatings exhibited a variable frictional moment in the range of 180–260 N·mm, characterised by frequent and rapid fluctuations during time. These variations are typical for the dry friction of hard metal–ceramic coatings and indicate that sliding occurred through a cyclic adhesion–abrasion friction mechanism, an aspect also reported by Chowdhury MA. et.al. [55], who analysed the behaviour of metal–ceramic composites under dry friction. It can be suggested that the main causes that generate frictional moment variations within such wide ranges are as follows: (a) the repeated formation and degradation of a tribochemical oxide film, similar to the reports of Rukhande S.W. et al. [56]; (b) interactions with exposed ceramic particles (Cr_3_C_2_, WC, TiC, BC, etc.) causing friction impulses [57]; and (c) the presence of ceramic asperities that can generate intermittent microscopic welds followed by fracture, which generates large friction impulses, similar to those reported by Kekes D. et al. [58]. It can be stated that in this study, the friction is unsteady but not catastrophic and the coating withstands loads without obvious signs of collapse or rapid wear. The friction couple of the 97MXC coating exhibits a behaviour specific to very hard materials in dry contact, which tends towards slightly oscillating abrasive wear.

The frictional moment of the 60T/WC torque is lower, stable at around 140–180 N·mm, and its variation curve is smooth, with very few disturbances. The presence of tungsten carbide particles in the coating provides the surface with a high hardness, resistance to solder bonding, reduced adhesion, minimised microwelds, and possibly the formation of a tribologically stable tungsten oxide layer that reduces friction [58,59]. It can be suggested that this coating tends to slide by a controlled abrasive friction mechanism without significant adhesion.

**Figure 10:**
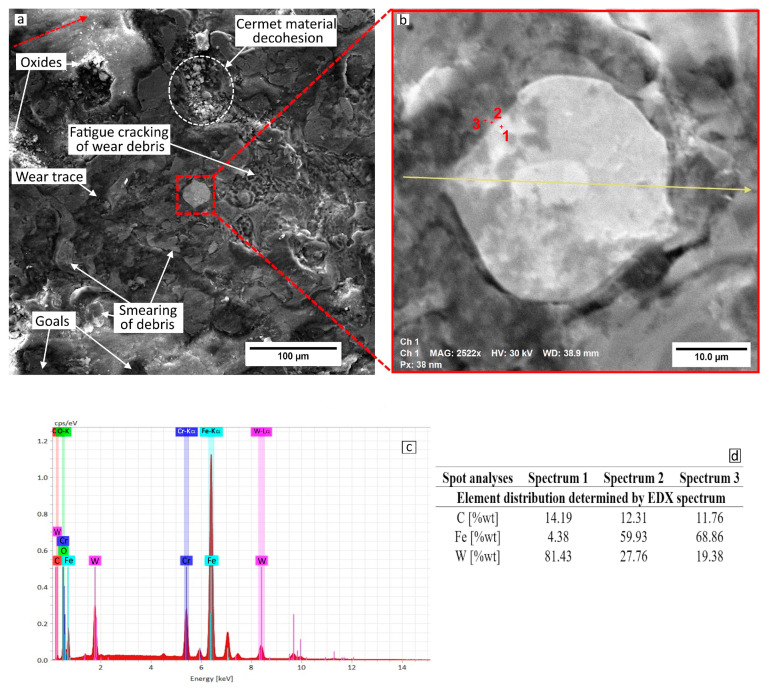
Surface damage of 60T/WC: (**a**) SE image of the wear trace; (**b**) WC—detail; (**c**) spectrum of chemical elements in the median zone of tungsten carbide; and (**d**) EDX spot analysis.

Measurements made using the roughness meter show that the wear traces and depth of the 97MXC and 60T/WC deposits recorded at a load of 40 N are equal to 380 ± 52 μm and 1.82 ± 0.12 μm and 420 ± 83 μm and 2.62 ± 0.22 μm. The main wear mechanism is abrasive grooving followed by fatigue that occurs between the wolfram carbide-containing deposit and the counter ball. In Figure 9 and Figure 10, micrographs of the wear channels of the 97MXC and 60T/WC deposits are presented, as well as some details regarding the method of embedding WC in the metal matrix. The greatest loss of material—which is obtained for the deposition of 60T/WC—is as a result of the action of the wear debris of the deposited layer.

It can be suggested that the debris was transferred and spread across the surface of the ball, behaving like a third body that damaged the friction torque. The fatigue phenomenon accentuated the decohesion of the material, influencing the stability of the carbides in the layer, detaching them under the action of mechanical load. The detached material was transferred through the wear track, and the wear debris accelerated the wear of the formed grooves and the wear of the deposits, respectively. It can be suggested that the adhesive transfer of debris through the wear track caused the deposits to deteriorate and the wear channels to become more pronounced. The favourable atmosphere (high oxygen and nitrogen content and high temperature due to friction) determined the occurrence of tribochemical reactions at the level of wear products and their subsequent entrainment in the initial scratches of the surface, with the formation of a tribofilm rich in wear debris. These suffered severe plastic deformations and finally caused the wear products to detach, resulting in material loss [60,61]. It can be observed that, in the 97MXC coatings, the WC particles were embedded in the iron matrix using a transition layer formed by a ferrite alloyed with W—see Figure 9d. In the 60T/WC deposits, the analysis of EDX spots at the WC particle–iron matrix interface—see Figure 10d—reflected the absence of an intermediate layer at the interface level. It can be suggested that, in the 97MXC deposits, tungsten carbide particles, melted in the electric arc, formed a transition layer capable of fixing the carbide inside the metal matrix at the interface with the metal matrix. The thickness of this layer depended on the technological process conditions [25] and explains the superior wear behaviour of the 97MXC coatings vs. the 60T/WC coatings.

## 4. Conclusions

The purpose of the investigations carried out in this work was to research the possibility of obtaining, by electric arc spraying, a coating of stainless steel reinforced with WC particles, resistant to wear and erosion by cavitation, using a spray nozzle system capable of introducing metal carbides in powdered form into the electric arc. We highlighted, evaluated, and compared the effects of microstructure and mechanical properties on the wear and erosive performance of 97MXC and 60T/WC materials coated on a C15 steel substrate. Therefore, the possibility of obtaining a 60T coating “doped” with WC was proven, symbolically called 60T/WC. An analysis of the results allows us to affirm that the EC performance of the coatings depended on the uniformity of the microstructure, while the wear resistance to dry friction was correlated with their mechanical properties. No relationship between the resistance to CE and dry friction wear was found. On the other hand, both coatings used in our research increased the anti-damage properties of the steel substrate. As a result of the research carried out, the following can be affirmed:

- The 60T/WC coatings presented an erosion by cavitation resistance 7.2 times lower than that of the C15 carbon steel sample, close to that of the 97MXC coatings, which offered a higher erosion by cavitation resistance;
- Both types of coatings presented relatively stable erosion rates, as well as similar values of average erosion depth (MDE), six times higher than the C15 steel sample;
- The wear resistance tests by dry friction demonstrated that, although the CoF of the 60T/WC coatings was more stable and presented values approximately 10% lower than the CoF of the 97MXC, the wear of the 60T/WC layers—recorded for 40N loads—was over 200% higher than the wear of the 97MXC coatings. This aspect demonstrates that the 97MXC coatings presented a better behaviour in response to dry friction than 60T/WC;
- The dominant wear mechanism was abrasive wear, followed by fatigue. Both phenomena were determined by the cohesion of the layer material and the weakening of the WC particles. The wear products formed a tribofilm subjected to plastic deformation and detached from the wear track. The wolfram carbides in the 97MXC coating were embedded in the metal matrix through an intermediate layer of ferrite alloyed with wolfram, which increased the wear resistance of the coating.

The frictional torque of the 60T/WC coating showed more stable dry friction behaviour compared to the 97MXC layer, showing less variation and less uncontrolled wear, respectively, making it an optimal coating for non-lubricating applications where stability and slow wear are priorities. The frictional torque of the 97MXC coating had a higher and unstable frictional moment, but this does not mean that it was weak; on the contrary, it was very resistant and could withstand high loads. The oscillations observed are typical for ceramic–metal materials in dry contact, but also indicate a higher risk of localised wear.

It can be suggested that although the 60T/WC coating had worse EC behaviour compared to the 97MXC coating, it can still be considered as a relatively good and cheap solution for protecting C15 steel parts.

In conclusion, this study substantiates the capability of electric arc spraying to produce WC-reinforced stainless-steel coatings, markedly enhancing the wear and cavitation erosion resistance of C15 steel substrates through mechanisms governed by microstructural homogeneity and intrinsic mechanical properties. While the 97MXC coatings demonstrated a superior tribological and cavitation erosion performance, 60T/WC coatings represent a cost-effective and structurally stable alternative for applications requiring reliable protection under dry and moderately abrasive conditions.

## Figures and Tables

**Table 1:** Chemical composition of the used materials.

Materials	Chemical Compositions (%)										
	C	Cr	B	Ni	Si	WC	P	S	TiC	Mn	Fe
**C15**	0.12–0.18	-	-	-	0.4	-	0.045	0.045	-	0.3–0.6	Balance
**97MXC**	-	13	2	6	1	26	-	-	6	-	Balance
**60T**	0.3	13	-	1	1	-	-	-	-	1	Balance

**Table 2:** Technological parameters.

Parameters	Value	
	97MXC	60T/WC
Current intensity (A)	240	180
Voltage (V)	32	30
Primary air pressure (bar)	6	6
Secondary air pressure—for WC training (bar)	-	2
Stand-off distance—SOD (mm)	180	200
Movement speed of the gun (m/s)	0.14	0.14
The number of passes	3	3

**Table 3:** The values of mechanical properties.

Coating	Porosity—According to ASTME 2109-01 [%] [35]	Adherence—According to ASTM C633-13 [MPa] [45]	HV_300_—According to ASTM E384 [49]
97MXC	11.42% ± 0.7	47.12 ± 4.1	823 ± 34
60T/WC	13.26% ± 0.5	42.76 ± 3.8	656 ± 42

**Table 4:** Results of erosions by cavitation of the C15 samples, with and without coatings.

	Sample Type		
	97MXC	60T/WC	C15
Total mass (mg)	87.4	75.6	77.32
Average erosion depth (μm)	24.2	28.7	328
Maximum erosion (μm/h)	5.7	12.4	96.2
Lost volume (mm^3^)	6.3	10.5	76.4

**Table 5:** Mean CoF and wear in wt%.

Parameters	Materials			
	97MXC		60T/WC	
Load [N]	20	40	20	40
CoF	0.122 ± 0.011	0.238 ± 0.024	0.110 ± 0.028	0.215 ± 0.012
Wear [g/h]	0.002 ± 0.008	0.029 ± 0.015	0.043 ± 0.018	0.089 ± 0.021
Initial mass [g]	42.276	41.886	41.736	42.273
Final mass [g]	42.274	41.854	41.681	42.181

## Data Availability

The original contributions presented in this study are included in the article. Further inquiries can be directed to the corresponding authors.
